# Asymmetry in Food Handling Behavior of a Tree-Dwelling Rodent (*Sciurus vulgaris*)

**DOI:** 10.1371/journal.pone.0118233

**Published:** 2015-02-25

**Authors:** Nuria Polo-Cavia, Zoraida Vázquez, Francisco Javier de Miguel

**Affiliations:** 1 Department of Biology, Universidad Autónoma de Madrid, 28049, Madrid, Spain; 2 Department of Biodiversity and Evolutionary Biology, Spanish National Museum of Natural History (CSIC), 28006, Madrid, Spain; University of New England, Australia, AUSTRALIA

## Abstract

Asymmetry in motor patterns is present in a wide variety of animals. Many lateralized behaviors seem to depend on brain asymmetry, as it is the case of different tasks associated to food handling by several bird and mammal species. Here, we analyzed asymmetry in handling behavior of pine cones by red squirrels (*Sciurus vulgaris*). Red squirrels devote most of their daily activity to feeding, thus this species constitutes an appropriate model for studying asymmetry in food processing. We aimed to explore 1) the potential lateralization in handling of pine cones by squirrels, 2) the dominant pattern for this behavior (left- vs. right-handed), and 3) whether this pattern varies among populations and depending on the pine tree species available. Results revealed that red squirrels handle pine cones in an asymmetrical way, and that direction of asymmetry varies among populations and seems to be determined more by local influences rather than by the pine tree species.

## Introduction

Vertebrate animals commonly exhibit a preferential use of anatomical structures of one side of the body, which can be attributed to functional asymmetries between the two cerebral hemispheres. The right hemisphere is known to control functions such as early detection and escape from predators [1, 2], fear [3], courtship and copulation [4], visual recognition of conspecifics [5] and spatial cognition [6]. By contrast, functions attributed to the left hemisphere are discrimination and capture of prey, motor skills and recognition of typical conspecifics vocalizations [2, 7, 8].

Approaches to vertebrate laterality have largely analyzed the response to visual stimuli in species with monocular vision, due to their laterally placed eyes [5, 6, 9, 10]. Many of these studies have been performed on birds, demonstrating the existence of lateralized visual tasks [1, 6, 10]. Visual functions have also been studied in teleost fish, showing a clear correlation between lateralized responses and the eye involved [11]. Amphibians and reptiles have received less attention, but however, righting behavior of tortoises (i.e., the ability to turn the right side up when they result overturned accidentally, which is critical to survival) has been found to be skewed to the right [12]. On the other hand, aggressive responses of several species of frogs and lizards are strongly lateralized and controlled by the right hemisphere [8].

Certain social tasks and emotionally significant responses are also lateralized. For example, young calves of wild beluga whales (*Delphinapterus leucas*) swim and rest significantly longer on their mother’s right side, showing a left eye preference during calf-mother social interactions [13, 14]. Similarly, human and non-human primates exhibit a left-side bias in infant cradling [15, 16]. In a recent study [17], great apes (*Pan troglodytes* and *Gorilla gorilla gorilla*) tended to keep conspecifics in the left visual field, which is widespread among vertebrates, pointing to a common evolution of right-hemisphere dominance for social responses [18]. Also, ring-tailed lemurs (*Lemur catta*), both in captivity and in the wild, leave olfactory marks preferably with the left forearm [19], and domestic horses (*Equus caballus*) show different laterality patterns according to emotional value of objects inspected [3]. However, not all experiments support presence of laterality. For example, goldbelly topminnows (*Girardinus falcatus*) do not exhibit preferential side escape in response to a simulated predator attack [20]. Likewise, lateralization has not been observed in newborn lambs when they lie down, wags its tail when sucking or initiate gait [21]. Moreover, several studies on New Caledonian crows (*Corvus moneduloides*) show no consensus about presence or absence of laterality in tool use [22].

Among lateralized behaviors, feeding can be considered of critical importance, since animals must perform certain motor acts to optimally obtain and process the food providing the energy intake necessary for their daily activities. Lateralized foragers can optimize foraging by reducing handling time, thus diminishing the risk of predatory attacks during feeding and ultimately increasing fitness [23–25, 26]. For example, completely right-lateralized chimpanzees (*Pan troglodytes*) are more efficient (i.e., gather more prey per unit effort) than incompletely lateralized individuals [27]. In birds, greater ability of the left hemisphere to recognize food has been demonstrated in pigeons (*Columba livia*), chickens (*Gallus gallus*) and zebra finches (*Taeniopygia guttata*) [9]. On the other hand, preference for the left hind limb (right hemisphere) in handling food has been described in Australian parrots [28, 29], and similar results have been obtained recently in the Kramer’s parakeet (*Psittacula krameri*) [30]. Asymmetries in foraging can be useful also in detecting predators during feeding. For example, right jaw dominance would allow retaining unimpeded vision from the left eye for vigilance tasks while eating [2, 31–35]. Thus, laterality in food handling behavior might have evolved under the influence of adaptation to a predation environment. An extraordinary case of lateralized feeding behavior is found in the New Zealand wry-billed plover (*Anarhynchus frontalis*), which is the only bird in the world whose beak is twisted to the right. This morphology has evolved as an adaptation enabling the plover to employ the right eye in seeking its food under river stones [36, 37].

Regarding rodents, there are references to both left- and right-handed squirrels in relation to the way they handle pine cones in field guides of animal tracks [38, 39], but systematic research covering this topic has not been conducted until now. Squirrels are excellent models to test for asymmetry in food handling behavior, given that they are essentially forest arboreal animals that spend much of their time budget searching for and consuming food. Their diet is based on a high caloric intake that includes berries, buds and small animals, but mainly nuts and pine seeds from cones of several pine tree species [40]. Since pine seeds are packaged inside pine cones, their extraction is time-costly. Hence, handling behavior of pine cones by squirrels is expected to be lateralized. Lateralization could be also influenced by differences in hardness or even in bract structure of cones between *Pinus* species; the difficulty extracting seeds from cones being decisive in the evolution of handling asymmetries. This is the case of several non-human primates, for which the strength of manual preference increases with the complexity of the task (i.e., tasks involving low manipulative demands lead to symmetrical distributions of hand biases whereas high complex behaviors requiring specialization and bimanual coordination show asymmetrical patterns) [41, 42]. Hence, asymmetries in handling behavior of cones by squirrels might be related to dissimilar task complexity posed by different pine tree species. In this paper we explore the potential lateralization in extraction of seeds by red squirrels (*Sciurus vulgaris*), the dominant trend, and its relation to the collection site and the *Pinus* species. Specifically, we analyze: 1) whether handling behavior of pine cones by squirrels follows an asymmetrical pattern, 2) whether squirrels are predominantly left-handed or right-handed in food handling, and 3) to what extent a potential left/right handling bias varies among populations and depending on the pine tree species available.

## Material and Methods

### Handling Behavior of Pine Cones

**Fig 1:**
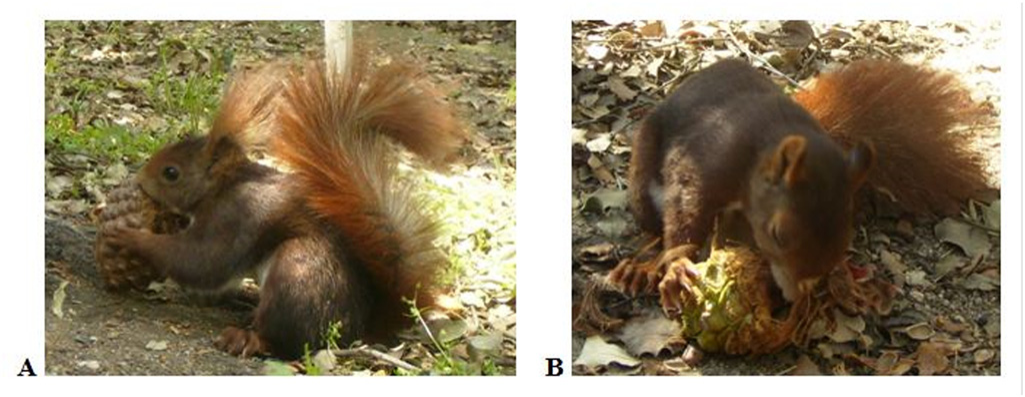
Red squirrel handling a pinecone. A: starting to gnaw the cone. B: supporting the cone horizontally, with the apex pointing to the right side.

**Fig 2:**
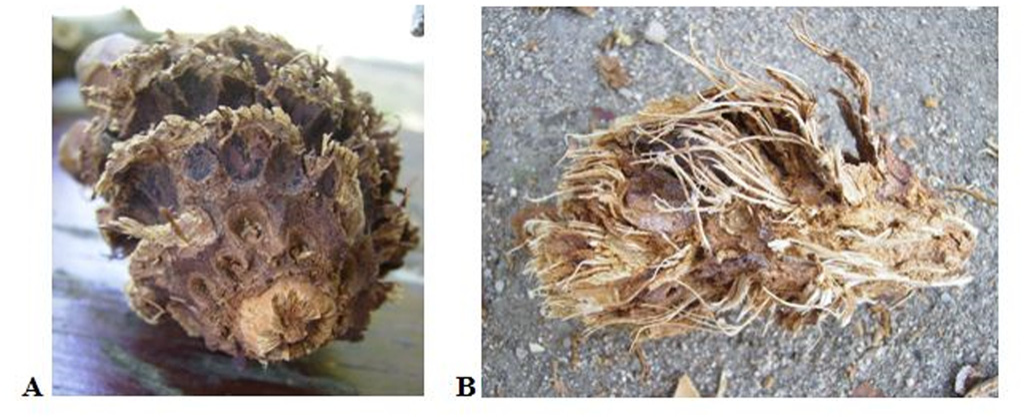
Pinecones handled by red squirrels. A: ‘right’ cone. B: torn cone.

The preliminary phase of the study consisted in direct observation of feeding behavior of red squirrels at the Breeding Centre of Red Squirrel in Casa de Campo (Madrid Province, Central Spain). This center is located in an extensive, suburban holm oak forest. Two pairs of red squirrels were housed in separate enclosures (8 x 8 x 6.5 m) equipped with feeders, drinkers and nest boxes (although squirrels often build their own nests), and habitually fed nuts, apples, carrots and pine cones. For the sessions, squirrels were supplied Aleppo (*Pinus halepensis*) or stone (*Pinus pinea*) pine cones collected in the field. Observations were distributed in 18 sessions from May to July 2010. Handling behavior of cones by squirrels was registered through pictures and videos and recorded in field notebooks. From direct observations of squirrels’ feeding behavior at the Breeding Center, we drew out a basic pattern of their handling of pine cones that we used later to accurately interpret lateralization of this behavior from gnawed cones found in the field. Thus, we noticed that squirrels started to gnaw pinecones at their base, holding them vertically on their apex (Fig. 1A). In this way, squirrels removed the pine scales at their base, often leaving a small ridge in the site of the extraction. Then squirrels placed the cone horizontally, holding the base with one of the forelegs and the apex with the other (Fig. 1B). This latter is used to revolve the cone, while gnawing the bracts. The action of the lower incisors on the bracts leaves an oblique track on the cone with the shorter edge closer to the hemimandible and the longer edge in the opposite side. Looking at the disposition of this track in gnawed cones collected in the field (Fig. 2A), we could distinguish between left-handed squirrels, which hold the cone with the apex towards the left (i.e., use the left hand to rotate the cone), and right-handed squirrels, which do the opposite. We assumed no bias between left- and right-handed squirrels in the proportion of cones they ate.

### Study Sites

Red squirrels are common in mountain and forest areas in Madrid Province, as well as in several urban and suburban parks of the city, where the basis of its feeding, mainly pine seeds, is easy to find. Hence, we collected gnawed pine cones by squirrels in four sites within Madrid Province where different *Pinus* species are present: 1) ‘Dehesa de la Villa’ (40°27’ N, 3°43’W; *n* = 139), urban park with *P*. *halepensis* and *P*. *pinea*, 2) ‘Casa de Campo’ (40°25’ N, 3°45’W; *n* = 81), suburban forest of mainly *P*. *pinea*, 3) ‘Valdelatas’ (40°32’ N, 3°41’W; *n* = 236), peri-urban forest of *P*. *halepensis* and *P*. *pinea*, and 4) ‘Valdilecha’ (40°17’ N, 3°18’W; *n* = 437), forest of *P*. *halepensis*. Cones were collected from 3 or 4 areas (30 x 30 m) within each site, and we averaged data from these areas considering each site as a single sampling unit in order to eliminate the effects of replication and pseudo-replication. Collecting sessions took place from October to November 2010, once every 15 days (4 sessions in total). Collecting effort was equal for all the areas (i.e., we spent the same time collecting in each area and collected all the cones that we could find within). We ensured that collected cones had been eaten by squirrels and not by other animals by consulting field guides of animal tracks and through careful observation of cones eaten by squirrels at the Breeding Center. Cones were classified as ‘left’ or ‘right’ basing on the presence of clear directional gnawing tracks that allowed us to distinguish them unequivocally. When we appreciated both left and right gnawing tracks on the surface of a cone in such a way that no clear pattern was evident, the cone was discarded. Non-clear asymmetric cones and torn cones (Fig. 2B) represented 13.4% of total cones collected and were not used in the analysis. No specific permissions were required for collecting pine cones at the locations and no endangered or protected species were involved in the study.

### Data analyses

Data from collected gnawed cones did not meet parametric assumptions, thus we used non-parametric tests to analyze the effects of collection site and *Pinus* species on lateralization of handling behavior of cones by red squirrels. We conducted a log-linear model to evaluate the effect of these variables and their interactions. A three-way contingency table was generated by the factors laterality (‘left’ vs. ‘right’), collection site (‘Dehesa de la Villa’ vs. ‘Casa de Campo’ vs. ‘Valdelatas’ vs. ‘Valdilecha’), and *Pinus* species (‘*P*. *halepensis*’ vs. ‘*P*. *pinea*’). Two-tailed binomial tests were used to assess differences in direction of laterality across collection sites and *Pinus* species, and differences in abundance of *Pinus species* across collection sites. All analyses were performed using Statistica software.

## Results

We collected a total of 893 pine cones, 773 out of which showed clear signs of asymmetrical gnawing (46.2% were left-gnawed, whereas 53.8% were right-gnawed) (Table 1). Asymmetric cones were more abundant in Valdilecha (53.4%), and belonged predominately to *P*. *halepensis* species (85%) (Table 1). Results of the fit of log-linear models to the three-way contingency table crossing the effects of laterality, collection site and *Pinus* species showed significant differences in pine cone frequencies for all of these variables and their corresponding interactions (Table 2). The effect of laterality was significant in all sites, although the direction of lateralization was not always the same: cones were predominately right-gnawed at Dehesa de la Villa (75/10; two-tailed binomial test, *p* < 0.0001), Casa de Campo (47/23; *p* = 0.006) and Valdelatas (179/26; *p* < 0.0001), whereas left-gnawed cones were more abundant at Valdilecha (115/298; *p* < 0.0001). Regarding the interaction between laterality and pine tree species, *P*. *halepensis* cones were significantly right-gnawed (356/301; two-tailed binomial test, *p* = 0.035), but there were no significant bias in the direction of lateralization for *P*. *pinea* cones (60/56; *p* = 0.78). *P*. *halepensis* cones were more abundant at Dehesa de la Villa (76/9; two-tailed binomial test, *p* < 0.0001) and Valdelatas (168/37; *p* < 0.0001). At Valdilecha we only found *P*. *halepensis* cones (*n* = 413), whereas at Casa de Campo *P*. *pinea* cones were only present (*n* = 70).

**Table 1:** Collection site, *Pinus* species and laterality (left vs. right) of gnawing for collected pine cones.

Collection site	*Pinus* sp.	Laterality	
		Left	Right
Dehesa de la Villa	*P*. *halepensis*	1	75
	*P*. *pinea*	9	0
Casa de Campo	*P*. *halepensis*	0	0
	*P*. *pinea*	23	47
Valdelatas	*P*. *halepensis*	2	166
	*P*. *pinea*	24	13
Valdilecha	*P*. *halepensis*	298	115
	*P*. *pinea*	0	0

**Table 2:** Results of the log-linear model for the three-way contingency table generated by the factors collection site, *Pinus* species and laterality.

Effect	*G* ^*2*^	*df*	*P*
Laterality	4.46	1	0.035
Collection site	365.7	3	0.001
*Pinus* sp.	412.62	1	0.001
Laterality x collection site	389.86	3	0.001
Laterality x *Pinus* sp	121.66	1	0.001
Collection site x *Pinus* sp.	507.44	3	0.001

## Discussion

Our study evidences the existence of asymmetry in handling of pine cones by red squirrels, both from direct observations of individuals at the Breeding Center and from statistical analyses of data from the pine cones collected. Careful observation of squirrels’ handedness during feeding in the preliminary phase of our study allowed us to establish objective criteria for a proper interpretation of the indirect rests collected in the field, which contain essential information relating to handedness of animal motor skills and its relevance as an advantageous adaptation. Squirrels, like other animals, are expected to adjust their foraging strategies in order to reduce predation risk and intra-specific competition during feeding, whereas maximizing energy intake [23–26]. This relies critically on quick handling of food [24, 25, 43]. Hence, the asymmetry in motor patterns we observed in the handling of pine cones by squirrels might be evolved as an adaptation to reduce handling time, increasing feeding efficiency and allowing saving time for other activities.

Asymmetry in feeding behavior is clearly functional in some animals, and can even affect feeding structures [44–47]. As such, asymmetry in handling of pine cones by squirrels might be related to body asymmetries conferring potential adaptive advantages. For example, hemimandibles can be unevenly mineralized (i.e., skeletal asymmetries), and thus attacking the pine cones with the more mineralized jaw would be advantageous. In humans, it has been observed that hard foods evoke more masticatory laterality [48]. This could be also the case for squirrels, which commonly feed on foods requiring high munching efforts to be eaten. In fact, rostral stress generated during gnawing is much more intense in squirrels than in rodents with softer diets like guinea pig (*Cavia porcellus*) or brown rat (*Rattus norvegicus*) [49].

Pine cones collected in our study were mostly right-gnawed, suggesting a population bias in handedness of foraging behavior. These preferences would be presumably governed by cerebral asymmetries, with each side of the brain controlling different functions. Thus, the relatively simple task of holding a cone could be done with the left hand, whereas for the more complex task of rotating it, it would be better to use the right hand (controlled by the left side of the brain, which involves tasks requiring greater precision). On the other hand, asymmetrical handling of pine cones could be also influenced by the trade-off between foraging and vigilance tasks. It is well documented that mammals and other vertebrates rely preferably on the left eye for vigilance tasks [2, 31–35]. Consequently, right-handed squirrels attacking pine cones with the right hemimandible could benefit from keeping the left eye free to continue scanning the environment while eating. Supporting this, it has been demonstrated that grey squirrels (*Sciurus carolinensis*) are vigilant when they handle food in a semiupright posture, but that vigilance may be sacrificed when it compromises foraging [50].

Both the collection site and the pine tree species had a significant effect on lateralization of foraging behavior of squirrels. Regarding pine tree species, *P*. *halepensis* cones collected in our study were mostly right-gnawed, whereas no bias in the direction of laterality was found for *P*. *pinea* cones. This may lead us to believe that lateralization of feeding behavior in squirrels could be influenced by differences in the structure of pine cones between *Pinus* species. For instance, *P*. *halepensis* cones from Iberian Peninsula are oblong-conical (6–12 cm long, 3.5–4.5 cm wide) with slightly convex bracts. Conversely, *P*. *pinea* cones are rather oval-globular (8–15 cm long, 7–10 cm wide) and present clearly convex bracts [51]. These differences might explain the absence of hand bias found in gnawed *P*. *pinea* cones. In fact, squirrels very often attack *P*. *pinea* cones on rocks or stumps in the ground, employing a procedure to extract the seeds that is not observed for cones of other pine tree species [52]. Besides, pine cones contain unequal numbers of clockwise and counter-clockwise acropetal generative spirals, and the number of spirals differs among pine species [53]. Thus, handling asymmetries exhibited by squirrels in our study might also reflect a way to deal with naturally asymmetrical pine cones. However, considering data overall, it seems that direction of lateralization is more related to collection site rather than to *Pinus* species, suggesting a phylogenetic effect on handling bias within squirrel populations. In fact, right-handed squirrels prevailed in three out of the four locations we studied (Dehesa de la Villa, Casa de Campo and Valdelatas). Dehesa de la Villa and Valdelatas show very similar characteristics in pine cone sp. availability (i.e., both *P*. *halepensis* and *P*. *pinea* are present) and most collected pine cones were right-gnawed. In Casa de Campo, collected pine cones were also mainly right-gnawed, but only *P*. *pinea* is found in the sampled area. By contrast, in Valdilecha, where pine forest consists exclusively of *P*. *halepensis*, collected cones were mainly left-gnawed.

Unfortunately, we do not know the relationship between our study populations, or whether squirrels from Valdilecha constitute a substantially different separated genetic nucleus. In absence of such information, the phylogenetic or adaptive nature of the differences in handling bias we observed among populations cannot be elucidated.

Alternatively, handedness in feeding behavior has been shown to temporally change within populations, being the abundance of left- and right-handed individuals regulated by frequency-dependent natural selection. So, it has been demonstrated that the direction of mouth-opening in scale-eating cichlid fish of Lake Tanganyika (*Perissodus microlepis*) oscillates periodically every 5 years in response to frequency-dependent selection exerted by prey’s alertness [44]. Also, the ratio of bill crossing morphs of crossbills (*Loxia curvirostra*), which as squirrels eat pine seeds and rely on their asymmetric bills to extract them from the pine cones, seems to be the result of frequency-dependent selection, thus minimizing the overlap in the use of resources and enhancing foraging efficiency [45]. Likewise, lateralization in food handling behavior of squirrels might be determined genetically, and the ratio of handedness within the populations could vary over the evolutionary time according to frequency-dependent adaptations that maximize foraging. After all, individual brain efficiency is not necessarily related to the direction of lateralization of other individuals. At this point, Vallortigara and Rogers [8] stated that genes determine lateralization at the individual level, whereas developmental mechanisms align the direction of lateralization in the populations. Other previous studies manipulating steroid hormones have raised also the importance of genetic and environmental interaction, but suggest that environmental factors can only influence the degree of lateralization, whereas the direction of lateralization is determined by genetic factors [54–56]. For now, research at this point is not yet conclusive [57–58].

In short, red squirrels commonly handled pine cones in a lateralized way, being right-handed individuals (i.e. those holding the cone with the apex towards the right) apparently more common. Such lateralization might be associated with adaptive morphological and/or brain asymmetries favoring foraging efficiency and the outcome of a trade-off between feeding and vigilance. Nevertheless, the differences in handling bias we found among squirrel populations and related also to the species of consumed pine cones suggest, in agreement with previous studies, that both genetic and environmental components could be influencing the direction of laterality within the populations.
